# Cognition is entangled with metabolism: relevance for resting-state EEG-fMRI

**DOI:** 10.3389/fnhum.2023.976036

**Published:** 2023-04-11

**Authors:** Michael Jacob, Judith Ford, Terrence Deacon

**Affiliations:** ^1^Mental Health Service, San Francisco VA Healthcare System, San Francisco, CA, United States; ^2^Department of Psychiatry and Behavioral Sciences, Weill Institute for Neurosciences, University of California, San Francisco, San Francisco, CA, United States; ^3^Department of Anthropology, University of California, Berkeley, Berkeley, CA, United States

**Keywords:** EEG-fMRI, resting-state, neurovascular coupling, embodied cognition, psychiatric illness

## Abstract

The brain is a living organ with distinct metabolic constraints. However, these constraints are typically considered as secondary or supportive of information processing which is primarily performed by neurons. The default operational definition of neural information processing is that (1) it is ultimately encoded as a change in individual neuronal firing rate as this correlates with the presentation of a peripheral stimulus, motor action or cognitive task. Two additional assumptions are associated with this default interpretation: (2) that the incessant background firing activity against which changes in activity are measured plays no role in assigning significance to the extrinsically evoked change in neural firing, and (3) that the metabolic energy that sustains this background activity and which correlates with differences in neuronal firing rate is merely a response to an evoked change in neuronal activity. These assumptions underlie the design, implementation, and interpretation of neuroimaging studies, particularly fMRI, which relies on changes in blood oxygen as an indirect measure of neural activity. In this article we reconsider all three of these assumptions in light of recent evidence. We suggest that by combining EEG with fMRI, new experimental work can reconcile emerging controversies in neurovascular coupling and the significance of ongoing, background activity during resting-state paradigms. A new conceptual framework for neuroimaging paradigms is developed to investigate how ongoing neural activity is “entangled” with metabolism. That is, in addition to being recruited to support locally evoked neuronal activity (the traditional hemodynamic response), changes in metabolic support may be independently “invoked” by non-local brain regions, yielding flexible neurovascular coupling dynamics that inform the cognitive context. This framework demonstrates how multimodal neuroimaging is necessary to probe the neurometabolic foundations of cognition, with implications for the study of neuropsychiatric disorders.

## Introduction and motivation

Neuroimaging methods, such as functional magnetic resonance imaging (fMRI), provide relatively precise anatomical assessment of brain activity, but rely on slow changes in the vascular oxygen signal to infer information processing. By contrast, electrophysiologic methods (such as electroencephalography, EEG) depend on electric fields generated by the brain and provide high resolution temporal dynamics, but with relatively limited anatomical resolution. Simultaneous EEG-fMRI has developed as an approach to bridge the high temporal resolution of EEG with the spatial precision of fMRI. However, less work has considered how EEG-fMRI might be applied to investigate the coupling of vascular and metabolic signals with neural activity. Simultaneous EEG-fMRI might help reconcile differences between electrophysiologic and vascular/metabolic perspectives on information processing.

That the brain is an “information processor” is all but assumed. Information theory and related computational tools have advanced knowledge about stimulus-activity relationships from single neurons up to population-level activity (Quian Quiroga and Panzeri, 2009; Dimitrov et al., 2011). This methodological definition of neuronal information is based on the principle that numerical changes in neural data streams correlate with extrinsic manipulations (commonly referred to as the “neuron doctrine;” Yuste, 2015). The correlated change in neural activity is presumed to encode information about some attribute of the correlated stimulus and is modeled on computation. But both Turing's (1936) formalization of a general theory of computation and Shannon's (1948) mathematical theory of communication are agnostic about how these processes might be physically instantiated and the energy required to perform them. A digital computing device is therefore designed so that variations of its material components and fluctuations of the energy of its operations have essentially no impact on the formal properties of the computations it performs and the meanings that are assigned to it.

In contrast, information processes within a nervous system radically diverge from these computational criteria. Neuronal function is susceptible to being modified by changes in the local chemical milieu as well as in response to signals from other neurons. In particular, neurons are highly susceptible to changes in their metabolic support (Moreno et al., 2013; Iadecola, 2017). A neuron's functioning depends on the constant work of molecular pumps to maintain an ionic gradient across its surface membrane despite rapidly varying activity levels. And each time a neuron generates an action potential to initiate the propagation of a signal there is a significant energetic recovery required to prepare for the next. So the induction of rapid bursts is often followed by refractory periods during which this potential is re-established. The tight correlation between regional neural activity levels and local blood oxygen delivery is, of course, the rationale for treating the BOLD signal in fMRI as a surrogate for functional localization of brain functions (Mishra et al., 2021).

When we analogize neural function to logical circuit operations in computers, we implicitly (or even explicitly) assume that the energy is supplied irrespective of the content of information processing. Each operation to flip the charge of a semiconductor element during computer operation takes roughly the same amount of energy. So local energy use (often reflected in the heat of some component) is directly correlated with operations per second. The supply of energy to a computer thus plays no role in the structure of the operation being performed and is entirely determined by mere quantity of signal processing. We suggest that the distinction between computation and biological information processing depends on metabolism and that multimodal neuroimaging paradigms, particularly EEG-fMRI can identify the unique role of neurometabolic coupling for cognition.

Computational assumptions about brain processes have led to the so-called “dark-energy” problem of the brain. This problem asks why the brain utilizes a large amount of energy at rest, in the apparent absence of any cognitive processes (Carhart-Harris and Friston, 2010; Zhang and Raichle, 2010; Capolupo et al., 2013). But resting metabolic activity in the brain is only “dark” from the perspective of non-living systems; computers don't require energetic input at “rest” or when “asleep.” Resting metabolism in the brain is not merely for the support of living processes, it also supports incessant neural signal processing. The brain at rest is still incessantly active, processing information in background, whether awake and unfocused or in a dreamless sleep. Background activity is always present, even though obscured by a peripherally evoked significant increase in activity. Though it is often treated as background noise, this is too simple. To the extent that the structure of this “background” activity reflects intrinsic local circuit biases, it provides “self-in-context” information with respect to which non-self information can be juxtaposed.

So in general, the field of neuroscience currently does not make a formal distinction between the energetics of biological information processing and the energetics of computational information processing. Nonetheless, that metabolism is intertwined with information processing is increasingly recognized by models of neuronal intracellular energy homeostasis (Watts et al., 2018; Vergara et al., 2019), metabolic resource constraints (Laughlin, 2001; Burroni et al., 2017; Fardet and Levina, 2020), cognitive function (Collell and Fauquet, 2015), and consciousness (Pepperell, 2018). Whereas, these models suggest that it is likely that the energy use in brains plays a direct role in processing information, regarding the self/non-self pragmatic distinction, there is no counterpart in traditional computation. In what follows, we offer a view of embodied cognition that is dependent on the entanglement between metabolism and signal processing, highlighting how simultaneous EEG-fMRI might be used to investigate these interrelations.

## Entangled embodied cognition

**Figure 1:**
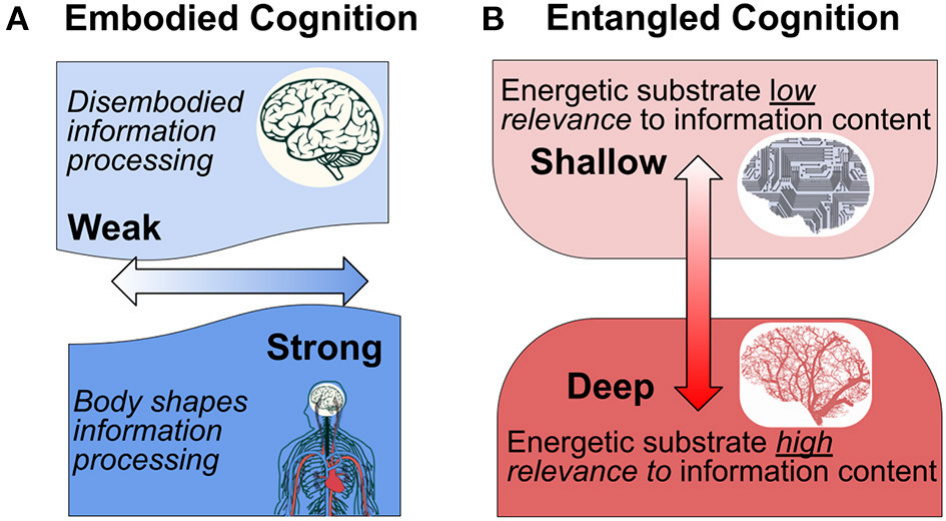
Interpretation of embodied cognition in the context of the nervous system. **(A)** Traditional conceptions of embodied cognition results are interpreted along a weak-strong horizontal axis (light to dark blue). According to the traditional interpretation, the “strength” of embodiment reflects the extent to which the body shapes cognition through a unified perceptual-action-cognition system (After Binder and Desai, 2011, who propose an intermediate “embodied abstraction” interpretation. According to their proposal, the impact of embodied semantics on perceptual representations depends on the context). **(B)** We hypothesize an orthogonal interpretation of “entangled” embodiment that indicates the extent to which the physical-energetic substrate is of relevance to information processing content.

The paradigm of embodied cognition has challenged the classical perspective that neural computations are logical operations that just happen to be processed by a living medium. The so-called “4E-cognition” approach considers that cognition is embodied, embedded, enactive, and extended (Newen et al., 2018). This paradigm recognizes that the context in which the organism is embedded, including both its organismic and ecological contexts, fundamentally shapes cognition. We propose a related interpretation of the role of embodiment in cognition based on the ways that neurometabolic energetics is entangled with signal processing in the brain (see Figure 1).

From this perspective we distinguish “shallow” from “deep” information processes with respect to the degree of entanglement between the patterns of signal generation, transmission, and modification, including the substrate in which these activities take place. In a computer, the energetics of signal processing is minimally relevant (if at all) to what the information is about. In this respect the information is “shallow” because it has nothing to do with its embodiment. In the terminology of functionalism, it is multiply realizable. The outside user/observer can effectively ignore the details of its physicality, except in the case that these properties fail to remain within the operational limits of the system and cause it to fail its design purpose.

By contrast, “deep” entanglement characterizes information processes in which the details of information value and use are inseparably entangled with the material and energetic processes that enable them. This is obvious with respect to information processes at the genetic and epigenetic levels, where specific molecular affinities and energetic relationships play critical roles in determining what takes place. This is because the specific physical and chemical properties are of direct relevance to cellular and organism function, and their interaction with the world (Gilbert, 2014; Bongard and Levin, 2021; Deacon, 2021). Analogously, we argue that the material and energetic embodiment of neural signal production and modulation cannot be disentangled from the information processing that constitutes cognition and mental experience.

To illustrate the distinction between “shallow” and “deep” entangled-embodied cognition, consider a non-biological example: a hand-held metal detector. A metal detector transmits an electromagnetic field from its search coil that can be disturbed by the presence of a conducting metal object (an extrinsic constraint). Detection of metallic objects is enabled by the parameters of the coil, the degree of sensitivity of the system, the work of generating the electromagnetic field, and of course, the positioning of the device in the environment. For both the metal detector and the brain, energy is required in order to operate. They both maintain far-from-equilibrium dynamics (maintenance of an electrical potential) that contribute to their capacity to do the work essential to their functions. In the case of the metal detector, the work of device maintenance is extrinsic; supported by an outside observer who also tunes the device to be sensitive to the physical properties that are relevant. Like a computer, the energy running the metal detector needs to only be stably supplied, sufficient to maintain the critical electronics, irrespective of the information it provides to the user. Unlike the metal detector, however, the brain processes information that is of intrinsic relevance to the system itself, including its specific physico-chemical constitution. The maintenance of far-from-equilibrium responsiveness in both systems requires thermodynamic work, accomplished by the same intrinsic, physical substrate that performs information processing. But for the brain there is no “outside observer” available to design, maintain, or interpret what the changing patterns of neural activity mean. Moreover, what is and is not meaningful to the brain involves its material and energetic constitution, not merely pattern matching or dynamical coupling. It is with respect to their embodiment that neural signals convey more than just physical patterns of activity that correlate with extrinsic patterns. As we shall review, metabolic embodiment is entangled in the incessant and ongoing “background” activity of neural circuits. This background activity is the focus of resting-state neuroimaging paradigms, so understanding the biological significance of this activity is of paramount importance.

**Figure 2:**
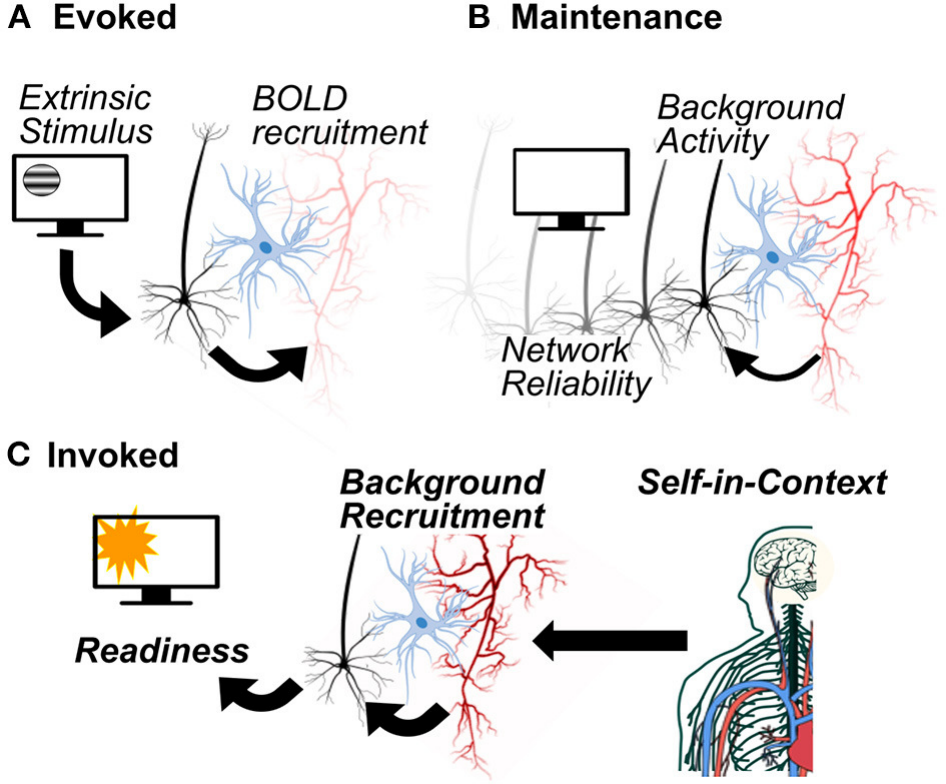
**Three** types of neurometabolic work to support cognition. Evoked work **(A)** is characterized by the recruitment of metabolic activity in response to stimulus driven neural activity. Maintenance work **(B)** does not depend on the presence of a stimulus: metabolic processes support neuronal background activity that, in turn, enables reliable synaptic network structure. Invoked work **(C)** anticipates the need for metabolic activity on the basis of the self-in-context, recruiting background activity for cognitive and behavioral readiness.

In order to characterize the impact of background activity on cognition, we will examine neurometabolism as reflective of three classes of work: (1) *e*voked work, (2) maintenance work, and (3) *in*voked work (Figure 2). Evoked work is the form of work typically studied in neuronal physiology and neuroimaging paradigms; linking increases in neurometabolic activity to specific cognitive processes, typically triggered by an extrinsic stimulus. In these evoked or event-related studies, background or baseline activity is subtracted from task activity (typical in the case of EEG-derived evoked potentials) or “removed” by statistical contrasts when comparing two related conditions (typical in the case of event-related fMRI parametric models). In either case, the goal is to isolate brain activation that is specifically evoked by the experimental variable under study, and where the background activity is not considered to be relevant. By contrast, maintenance work is not linked to extrinsic stimuli and instead reflects the physical-chemical work done to maintain circuit predispositions, supporting reliable synaptic network structure and resting-membrane potential (Laumann and Snyder, 2021). Sometimes referred to as “cellular housekeeping,” this form of work is typically not considered in neuroimaging paradigms although it reflects up to a third of neuronal energy use and sustains spontaneous background activity (Howarth et al., 2012). Lastly, we propose an additional category of work that is neither explicitly evoked nor merely “maintenance,” and reflects changes in neurometabolic activity that are intrinsic (not directly linked to a stimulus) and anticipate the regional needs of metabolic activity. By increasing (or decreasing) regional metabolic availability, invoked work “recruits” background activity. Increasingly identified from neuroimaging paradigms and animal studies, an invoked metabolic signal conveys additional information of contextual significance. Next, we will discuss these three classes of work in the context of cellular and systems neuroscience, including implications for simultaneous EEG-fMRI neuroimaging paradigms.

## Metabolic maintenance and background brain activity

According to the computing hardware-software analogy, the physical structures of neurons and networks are assumed to be the stage upon which the “actual” information processing occurs in the form of signal propagation (“shallow” embodiment). Others have pointed out that unlike a computer, living systems do not have clear hardware/software distinctions (Bongard and Levin, 2021). As such, computational models ignore the physical and energetic properties of the information processing medium (But see emerging paradigms, such as thermodynamic computing, Hylton, 2020; Hylton et al., 2021; and energy-aware computing; Kozma et al., 2018). By contrast, the brain does maintenance work to form and maintain the resting membrane potential, synaptic connections and network architecture. The presence of “spontaneous,” background activity within these networks enables maintenance of connections through hebbian mechanisms (Kirkby et al., 2013; Litwin-Kumar and Doiron, 2014). Thus, quite unlike the computational model, maintenance activity is necessary to sustain network organization. Referring to background activity as “spontaneous” is a misnomer, however. Thermodynamically speaking, it is decidedly *not*-spontaneous, it takes metabolic work. This metabolic work enables reliable synaptic network configuration, but raises a crucial question: is the ongoing, background activity merely epiphenomenal “noise” that sustains connectivity (akin to an idling engine in a car)?

Spontaneous background activity has long been recognized as critical to cortical development and the precise anatomy of brain circuit organization (Weliky and Katz, 1999; Blankenship and Feller, 2010; Molnár et al., 2020). Multiple lines of evidence now indicate that background activity in the mature brain is more than mere “noise” (Uddin, 2020). Historically, the presence of ongoing, spontaneous background activity in the absence of an experimentally defined stimulus or task was assumed to reflect random resting membrane fluctuations (Shadlen and Newsome, 1994; Amit and Brunel, 1997). Nonetheless, these fluctuations are correlated with trial-by-trial variability, suggesting that spontaneous activity and trial-by-trial correlations arise from similar biophysical processes (Kohn and Smith, 2005; Mendels and Shamir, 2018). Furthermore, although these fluctuations occur in the “background,” they impact psychophysical performance and behavior (Zohary et al., 1994; Faisal et al., 2008). Competing evidence has found that background correlations reduce statistical measures of information in animal studies involving perceptual descrimination (Bartolo et al., 2020; Kafashan et al., 2021). However, this reduction in statistical information coincides with successful behavioral performance, which in turn, is linked to greater reliability in activity across a population of neurons (Hennig et al., 2018; Valente et al., 2021). This seeming paradox between a reduction in statistical information and an increase in behavioral performance can be reconciled by the fact that statistical information in the above studies is determined relative to *extrinsic* stimulus-spiking relationships. Instead, background activity may convey contextual information relevant to behavior.

What is the nature of this contextual information? Notably, background activity demonstrates reliable stimulus “tuning” and response biases (Tsodyks et al., 1999). Spontaneous activity among co-active neurons show similar stimulus tuning preferences, and these same neurons are co-active during driven, extrinsic stimulus excitation (Grinvald et al., 2003; Omer et al., 2019). Additional studies have linked background activity to spontaneous motor behaviors in animals (Montijn et al., 2016; Stringer et al., 2019). These findings extend well-known effects of attention and alertness on background activity (Luck et al., 1997; Reynolds et al., 2000; Ecker et al., 2014). These contextual factors may be intrinsic, that is linked to spontaneous shifts of attention, arousal, and motor behavior, or additional extrinsic factors, as might be evoked by naturalistic environments (Berkes et al., 2011). Critically, ongoing activity forms the pre-stimulus “baseline” that modulates stimulus-evoked activity, perception and behavior. This has been observed in human (Boly et al., 2007; Hesselmann et al., 2008; Iemi et al., 2017; Podvalny et al., 2019) and animal studies (Quraishi et al., 2007; Gutnisky et al., 2017; Johnston et al., 2022).

In some sense, this background activity is similar to a metal detector insofar as it is “pre-tuned” (biased) to particular extrinsic constraints (stimulus preferences). However, in another sense, background activity in neurons is quite different from the metal detector, reflective of intrinsic organismal factors for which there is no machine analog. With respect to experimental paradigms, deviation from the expected background signal as a result of extrinsic stimuli or tasks, changes the relationship between background activity and the factors that caused the change in activity. However, this change in activity could also arise from intrinsic factors that may not be well captured by a simple additive process (as is typically modeled in neuroimaging paradigms). Non-stationarity in the background activity might corrupt statistical contrasts in fMRI models (Renvall et al., 2014; Evans et al., 2015) and baseline subtraction models in EEG (Wainio-Theberge et al., 2021). We now consider the impact of this perspective on neuroimaging paradigms more explicitly and the potential for metabolic signals to modulate background activity and contextual relevance.

## The resting-state paradigm is entangled with metabolism

Spontaneous brain activity in humans has largely been studied using the so-called “resting-state” paradigm. This paradigm describes a research context wherein participants are not given explicit cognitive or task instructions and are told to “rest.” (Some studies utilize an eyes open condition whereas others utilize an eyes closed condition and with varying specificity about what to do with one's thoughts). Therefore, brain activity generated under these conditions has been described as “spontaneous,” much like the spiking of individual neurons in the absence of an explicit stimulus. Concerns about the construct validity of “rest” and influence of resting instructions notwithstanding (Benjamin et al., 2010), resting-state activity ultimately provides the background context in which stimulus driven activity is embedded. Accordingly, there is more than mere analogy between the background electrical activity of single-neurons, which provides relevant local circuit context, and background activity across regional networks, which provides global “self-in-context” for the organism (Koban et al., 2021). Moreover, as was the case for background activity at the single neuron level, ongoing and background activity identified in neuroimaging paradigms is supported by high metabolic expenditure.

Although rhythmic resting-state activity has been studied for nearly a century using scalp-recorded EEG (Lennox et al., 1938), with the discovery of the default mode network (DMN) in the last 25 years and other so-called resting-state networks (Andreasen et al., 1995; Shulman et al., 1997; Gusnard et al., 2001a,b; Raichle et al., 2001), spontaneous activity from the resting-state paradigm has undergone a research renaissance. At rest the DMN shows robust metabolic activity and deactivates when participants engage cognitively demanding tasks (Raichle, 2015). The DMN is composed primarily of midline brain regions, including the medial prefrontal cortex and posteromedial regions including the posterior cingulate cortex, precuneus as well as posterior parietal cortex and the hippocampus. The midline regions of the DMN are the most metabolically active regions of the brain (Bleich-Cohen et al., 2012; Leech and Sharp, 2014). Of course, resting-state networks including the DMN were discovered from neuroimaging methods that rely on metabolic measures as a surrogate for neural activity, such as positron emission tomography (PET) and fMRI.

Resting EEG rhythms were historically interpreted and described as idling rhythms (Rhodes, 1969; Pfurtscheller, 1992), and the energy cost of this activity was not explicitly considered. Early multimodal neuroimaging studies demonstrated a to-be-expected, inverse correlation between thalamic glucose metabolism and alpha power (Larson et al., 1998), in line with an idling, lower energy state associated with resting-brain rhythms. Multiple simultaneous EEG-fMRI studies have identified correlations between resting alpha power and the default mode network (Scheeringa et al., 2012; Bowman et al., 2017; Marino et al., 2019). However, alpha-BOLD dynamics show considerable spatiotemporal variability (Mayhew and Bagshaw, 2017), overlap with multiple resting-state networks (Mantini et al., 2007) and are influenced by whether eyes are open or closed while at rest (Mo et al., 2013). Recent approaches to resting-state EEG have emphasized the relevance of broadband (aperiodic or non-rhythmic) activity (Donoghue et al., 2020) and that may be coupled to regions of the salience network (Jacob et al., 2021). Taken together, these findings suggest that resting brain activity (whether derived from EEG or BOLD signals) reflects active readiness embedded in the experimental context, rather than mere “idling.”

From the perspective of metabolic entanglement, this “readiness” may provide critical contextual information with respect to subject arousal and expectations. In the discussion above, we considered the background activity of single neurons as reflective of intrinsic contextual factors (e.g., spontaneous behavior and arousal). Intrinsic factors therefore provide a local context for extrinsically conveyed signals. By extension, the default mode network and other resting-state networks may play a similar role with respect to self-context. Both resting alpha and the DMN have been described as reflecting internal, self-oriented cognitive processing (Knyazev, 2013; Koban et al., 2021; Yeshurun et al., 2021). Moreover, both alpha and the DMN are linked to the embodied processing of autonomic signals and arousal (Beissner et al., 2013). These networks may reflect psychological self-content, such as narrative, autobiographical and episodic memory (Dastjerdi et al., 2011; Yeshurun et al., 2021) that is directly informed by autonomic arousal signals (Babo-Rebelo et al., 2016). Resting-state activity in the DMN has been proposed as a self-in-context dual-code; correlated with self-psychology and self-physicality (Koban et al., 2021). The deep entanglement perspective may offer an evolutionary explanation for this arrangement, in that the self-psychological context must always depend upon maintenance of a reliable self-physical or metabolic context. While this description offers a conceptual explanation of background and resting-brain activity, it does not address the possible impact that changes in metabolism may have on cognitive processing. Next we explore evidence for this possibility.

## Embodied context and the dynamics of neurometabolic coupling

In the sections above we considered that high levels of metabolic work enable reliable far-from-equilibrium activity in the background activity of single neurons, their populations and regional networks. This background activity supports the structural maintenance of network architecture in addition to providing a dynamic self-context for cognitive processing. Below we consider the extent to which changes in metabolism may directly or indirectly influence neural background activity and thereby contribute to different readiness contexts. Because any effect of metabolic change that modifies background activity originates internally, we refer to it as *invoked* work. The possibility of invoked work requires reconsidering whether metabolic processes are merely supportive of neural signaling, or whether they may impact or drive neuronal signaling directly (Watts et al., 2018; Vergara et al., 2019). This reconsideration has important implications for the design, implementation and interpretation of ongoing neuroimaging studies.

The proposal that changes in blood flow might actively facilitate background activity patterns is not new, having its origins in the so-called “hemo-neuro hypothesis” (Moore and Cao, 2008). In part, this hypothesis relates to many aspects of hemodynamic function that are unexplained by evoked work. For example, blood flow greatly exceeds the regional oxygen needs for active neurons (estimates suggest it may be as much as 20 times more) and has been described as “watering the entire garden for a single flower” (Ekstrom, 2021). Perhaps the brain errs on the side of excess blood flow, given the potentially devastating consequences of hypoxia. However, the overshoot effect suggests that the brain may have some flexibility to reallocate blood flow during stress (Elbau et al., 2018) as a compensatory mechanism in dementia (Becker et al., 1996) or in neuropsychiatric disorders such as schizophrenia (Tan et al., 2006). These examples raise the intriguing possibility that the overshoot may relate to the needs of “baseline” metabolic maintenance and intrinsic processes that are distinct from the extrinsic stimulus and evoked responses (Devor et al., 2011).

Maintenance of intrinsic signal responsiveness requires metabolic energy, and the proposal of invoked work suggests additional energetic processes that might further initiate or constrain electrical dynamics. Initially a source of controversy (Logothetis, 2010), multiple studies in animal models now support the hypothesis that metabolic energy might anticipate, regulate or initiate changes in neural dynamics. These include simultaneous intracellular work monitoring ATP in Drosophila (Mann et al., 2021), intracranial evidence combining functional ultrasound with LFP in rodents (Bergel et al., 2020), BOLD-LFP recordings in rhesus macaques (Schölvinck et al., 2010) and LFP-optical imaging methods in rhesus macaques (Sirotin and Das, 2009). Although the experimental context of the above studies varies, most point toward the relevance of task-related expectation and arousal, either with respect to a stimulus, task or state as driving hemodynamic activity (Cardoso et al., 2019). These studies are complemented by findings of flexible coupling and decoupling of neural and vascular signals, particularly during the transitions between active behavior and rest (Huo et al., 2014; Winder et al., 2017).

Basic cellular studies have identified subtle metabolic changes, such as mild hypoxia, that can modify neuronal excitability (Le Feber et al., 2018) and adaptive plasticity (Rybnikova et al., 2005). In fact, hypoxia may be typical in the developing brain, relevant to angiogenesis, neural network formation and the development of neurovascular coupling (Hillman, 2014; Kozberg and Hillman, 2016; Kozberg et al., 2016). In otherwise healthy human adults, the DMN specifically shows reversals of neurovascular coupling during mild hypoxia that are not seen in other brain regions, raising critical questions about the impact of these changes on cognition (Rossetti et al., 2021). Beyond oxygen status, food scarcity (Padamsey et al., 2022), change in neuronal fuel from glucose to ketone bodies (Ma et al., 2007) or circulating immune factors (Tonelli et al., 2005) can also impact neuronal information processing in animal models, highlighting the diversity of metabolic processes that may play a role in normal neural signaling (Watts et al., 2018). These experimental findings are further supported by computational efforts that suggest a role for bidirectional neurovascular coupling in the plasticity of neuronal selectivity (Kumar et al., 2019, 2021). Taken together, computational, cellular and systems level findings support the hypothesis that metabolic activity may serve as a generic mechanism to alter the context of information processing dynamics.

These findings complicate approaches that treat non-neuronal brain physiology as noise (Bright and Murphy, 2015; Das et al., 2021). Emerging evidence identifies synchronized activity within resting-state networks, including the DMN, that is driven by vascular stimuli (carbon dioxide inhalation) and in a manner that mirrors task-evoked networks (Bright et al., 2020). One interpretation of these findings is that the brain includes distinct neuronal and systemic “physiological networks” (Chen et al., 2020). However, as suggested by Bright et al., it is possible (and we might suggest probable) that these networks would interact, that is, vascular physiology may modulate or drive functional brain networks. This is supported by findings of hemodynamic signals preceding neural activity in humans from studies of EEG-fMRI in patients with epilepsy (Rathakrishnan et al., 2010) in addition to EEG combined with Near Infrared Spectroscopy (EEG-NIRS; Seyal, 2014). In healthy human participants, EEG-fMRI during the resting-state identified BOLD activity that precedes and predicts EEG rhythms across a wide range of EEG frequency bands and resting-state networks (Feige et al., 2017). Few studies have followed-up this intriguing result, and could be confirmed by re-analysis of existing EEG-fMRI datasets using lagged-correlation relationships, rather than conventional hemodynamic modeling. These findings are consistent with a growing literature that identifies variability in the shape or latency of the canonical hemodynamic response function (HRF; Rangaprakash et al., 2017; Elbau et al., 2018; Ekstrom, 2021). In fact, simultaneous EEG-fMRI has been proposed as an ideal method to help disentangle this variability in the hemodynamic response (Prokopiou et al., 2020) since the canonical hemodynamic response function was initially developed and has largely been studied in the context of event-related paradigms rather than during the resting-state. Alternative models, such as hemodynamic deconvolution, might also reveal unique resting-state hemodynamics (Wu et al., 2021).

## Multimodal approaches to study entangled cognition

Resting-state EEG-fMRI studies can offer an important avenue to investigate non-canonical, “invoked work” in models of neurometabolic coupling. Hemodynamic variability is also observed from multimodal neuroimaging that combines fMRI and PET-glucose metabolism in humans. This work has identified at least two distinct dynamic, neurometabolic coupling relationships: one for the DMN and another for the fronto-parietal (so-called, task-positive networks, Stiernman et al., 2021). Task positive neurometabolic coupling is characterized by canonical and lagged neurovascular temporal dynamics, typical of task evoked work. By contrast, regions of the DMN do not necessarily follow canonical hemodynamic coupling dynamics and are instead characterized by BOLD signal increases prior to task onset, followed by a negative response (Stiernman et al., 2021). In an accompanying commentary, Goyal and Snyder (2021) note that “why would the brain develop two relatively independent systems to engage in intrinsic vs. evoked activity is an important theoretical question.” We suggest that our characterization of evoked and invoked neurometabolic coupling offers a framework to address this question theoretically and experimentally. The findings of Stiernman et al., wherein BOLD activity increases prior to task onset, match prior findings of internally-driven preparatory task activity that has also been linked to the DMN (Goldberg et al., 2008; Soon et al., 2013; Sakata et al., 2017). Broadly, this preparatory activity is reminiscent of the readiness potential, or slow buildup of electrical potential prior to the onset of voluntary action, and that is typically measured using EEG. One intriguing possibility to arise from our proposed framework is that the slow-cortical potential, given that its timescale mirrors that of the BOLD response, may show near synchronous neurovascular coupling, and could serve a predictive or preparatory function (Khader et al., 2008; He and Raichle, 2009). That is, invoked work may reflect an entanglement between slow EEG rhythms and the BOLD response to set the readiness context through the modulation of background activity. Future EEG-fMRI work might investigate non-canonical neurometabolic coupling to EEG slow waves (<~0.1 Hz) that are linked to arousal (Toker et al., 2022), preparatory activity (Schurger et al., 2021) as well as fMRI defined functional connectivity metrics (Raut et al., 2021).

**Figure 3:**
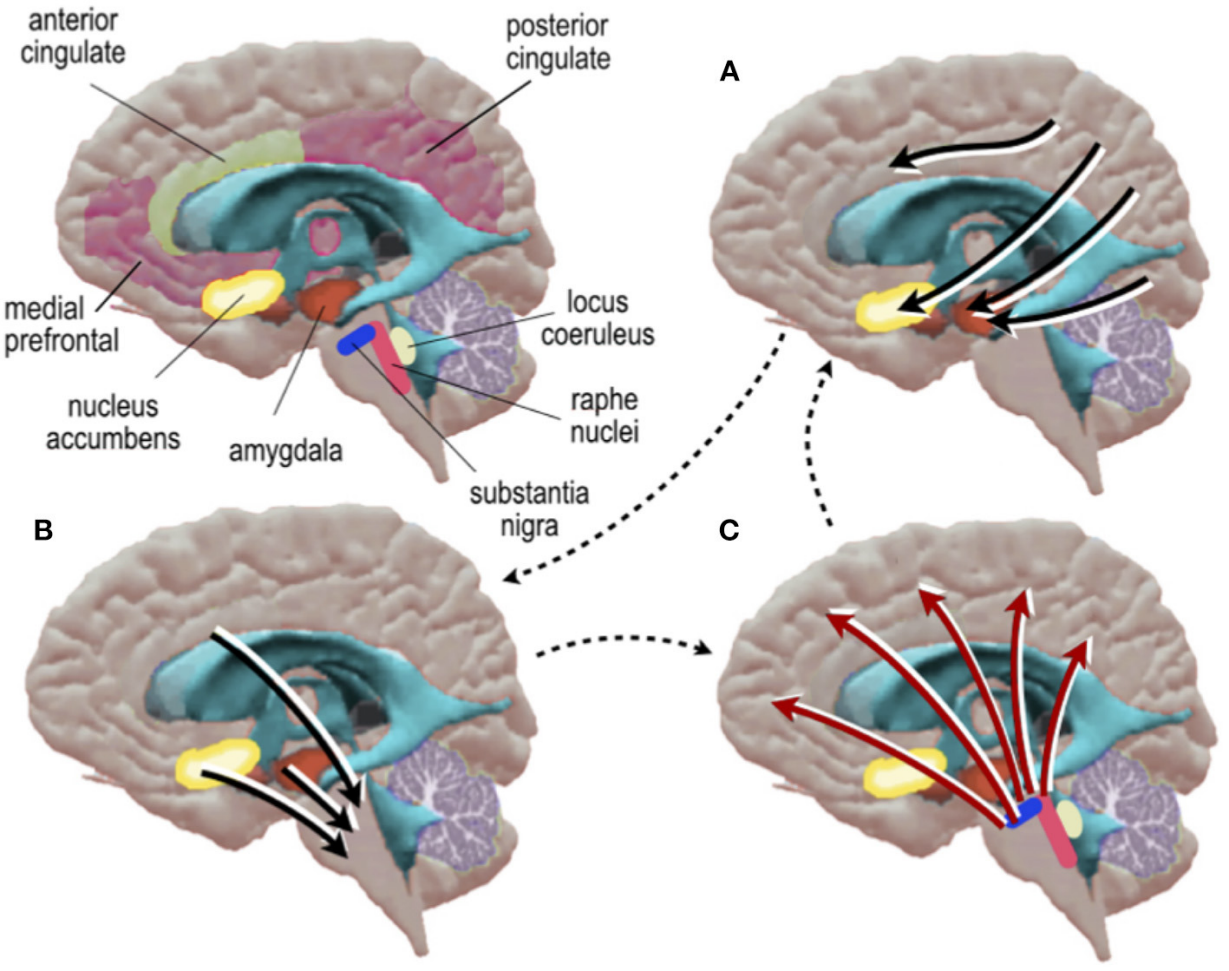
Schematic diagram of a hypothetical indirect neurometabolic circuit. **(A)** Cortical activity is assigned valence by basal forebrain nuclei which in turn, **(B)** modify the activity of neurons in midbrain-brainstem nuclei which in turn, **(C)** modify regional energy distribution in cortex.

What are the neural pathways that might support this invoked mode of work? Despite the evidence outlined above, few models have been proposed or experimentally studied. We suggest that at least two pathways may participate: a direct and indirect path (Figure 3). In the indirect path, certain patterns of neural activity, driven by biologically significant neuronal signals, modify background activity levels by up- or down-regulating local hemodynamic parameters. Specifically, brainstem and subcortical structures, which are tuned to biological context and are already known to mediate systemic and autonomic changes in arousal, are also known to mediate changes in regional brain metabolism and blood flow (Bekar et al., 2012; Toussay et al., 2013; Turchi et al., 2018; Özbay et al., 2019). Cortical afferents arising from the locus coeruleus and the basal forebrain have been found to shape low-dimensional energy landscapes, slow potentials and changes in awareness (Munn et al., 2021). Changes in the regional distribution of blood flow by arousal may, in turn, alter background activity patterns which will make the background activity in those regions more or less responsive to extrinsic input. This model is an extension of the explanation proposed by Elbau et al. (2018) to explain stress induced delays in metabolic coupling. In order to assay these subcortical and brainstem mechanisms, high field strength MRI is needed (Priovoulos et al., 2018). Emerging work suggests that simultaneous EEG-fMRI can be accomplished at this field strength (Jorge et al., 2015), offering an approach to test for evidence of an indirect path mediating cerebral blood flow *via* brainstem nuclei. In addition to this indirect (neural path), a direct (non-neural) path may also be of relevance, whereby peripheral or central metabolic factors, circulating in blood or in glial networks, might trigger changes in background neural activity (Ma et al., 2016).

Combining EEG with fMRI is a promising method to address the complexity of neurovascular and neurometabolic coupling. However, there are important limitations to this approach. EEG-fMRI is both technically and computationally challenging, given the artifacts generated by the magnetic field on EEG recordings (Allen et al., 1998, 2000; Fellner et al., 2016; Abreu et al., 2018). Moreover, EEG-fMRI has largely been focused on electrical-BOLD relationships. Measures of cerebral blood volume and flow, including distinguishing between arterial supply and venous drainage, are necessary to probe neurovascular coupling directly (Gao et al., 2017). Modeling the interrelationships between these vascular processes and neural activity is not straightforward, modulated by changes in behavior and at times, vascular physiology that is independent of neural activity (Drew, 2019; Das et al., 2021). Future studies might include EEG combined with arterial spin labeling, to examine electrical coupling to arterial supply (Mullinger et al., 2017; Zhu et al., 2017) or to changes in cerebral blood volume, as can be accomplished by measurement of Vascular-Space-Occupancy fMRI (Lu and van Zijl, 2012). Simultaneous studies of EEG and cerebral blood volume may be relevant in light of animal studies that have found a role for the locus coeruleus arousal system in mediating the coupling of cerebral blood volume with oxygen demand (Bekar et al., 2012). This finding is of relevance to our proposed hypothesis and for clinical conditions given evidence that acute and chronic stress impacts the molecular and cellular mechanisms of neurovascular coupling (Longden et al., 2014; Menard et al., 2017; Han et al., 2019, 2020). This basic work on neurovascular coupling may underpin clinical evidence for direct effects of metabolism on neuropsychiatric function (discussed below). Ultimately, convergent methods will be needed to examine neurovascular coupling from multiple hemodynamic measures including oxygen, flow, and volume to examine neurovascular coupling in human populations.

## Metabolism and embodied psychiatry

It is well known that psychiatric disorders suffer from so-called metabolic comorbidities, such as metabolic syndrome and diabetes (Penninx and Lange, 2018). These abnormalities are conventionally attributed to lifestyle changes and use of pharmacotherapy with metabolic side effects. However, genetic and “first-episode” studies have identified unique risk factors that may be predisposing in serious mental illness such as schizophrenia and bipolar disorder (Nielsen et al., 2021). These findings suggest that the entanglement between neural and metabolic factors may also unravel together during psychiatric illness. It has been suggested that the capacity and flexibility of human cognition comes at a high energetic cost (Navarrete et al., 2011) and comes with new vulnerability to energetic deficiency in the diet (Snodgrass et al., 2009). Given that recently evolved genes that enable metabolic efficiency are altered in serious mental illness (Khaitovich et al., 2008), neuropsychiatric disease may be related to specific cellular deficits in bioenergetic coupling and intermediary metabolism (Sullivan et al., 2018). In addition to intracellular abnormalities, serious mental illness is also linked to abnormal mitochondrial function and microvascular abnormalities that could impair all classes of work discussed above (Katsel et al., 2017; Whitehurst and Howes, 2022). Vascular and metabolic abnormalities might also underlie non-canonical hemodynamic responses seen across neuropsychiatric disorders (Ford et al., 2005; Hanlon et al., 2016; Lecrux and Hamel, 2016; Rangaprakash et al., 2017; Sukumar et al., 2020). These findings may complicate the use of resting-state paradigms that frequently identify abnormalities in the DMN in neuropsychiatric illness (Broyd et al., 2009; Whitfield-Gabrieli and Ford, 2012). New simultaneous EEG-fMRI investigations are needed that focus on neurometabolic coupling relationships, rather than studying neural or metabolic processes in isolation, in order to parse the impact of distinct neural and/or metabolic abnormalities.

In addition to clarifying the mechanisms of neural network function in psychiatric disease, an entangled cognition framework may enhance our understanding of novel interventions. Whereas, most psychiatric psychopharmacotherapy targets neural function, emerging approaches include dietary changes and initiation of physical activity to target cerebral metabolism (Firth et al., 2020). While glucose is normally considered to be the brain's default fuel, ketones bodies (derived from lipid metabolism) can provide an alternative fuel to the brain that is 27% more free energy than glucose (Sato et al., 1995). When individuals ingest a high-fat, low-carbohydrate, and adequate protein diet, the brain defaults to utilizing ketones for energy maintenance. There is a long history of using the ketogenic diet for refractory and pediatric epilepsy (Martin et al., 2016; D'Andrea Meira et al., 2019) and more recently, this diet has been studied in serious mental illness (Kraft and Westman, 2009; Bostock et al., 2017; Gilbert-Jaramillo et al., 2018). Neuroimaging studies have found that the increased neurometabolic efficiency of ketosis translates to greater network stability, as measured by sustained functional communication between regions from BOLD fMRI (Mujica-Parodi et al., 2020). Neuropsychiatric conditions, including serious mental illness and cognitive aging show inefficient use of cerebral resources; hypermetabolism at rest and hypometabolism during cognitive tasks (Potkin et al., 2009; Whitfield-Gabrieli et al., 2009; Oh and Jagust, 2013). Inefficient brain function could undermine maintenance work or the availability of resources for invoked work and therefore might benefit from metabolic interventions to improve efficiency. However, it can be difficult to ascertain adherence and/or the specificity of metabolic interventions on brain function given the wide range of dietary and exercise approaches (Rothman and Sheeran, 2021). Although the ketogenic diet can improve EEG markers of epileptiform abnormalities (Kessler et al., 2011), simultaneous EEG-fMRI is needed to directly assay neurovascular coupling and might be used to track the specificity of metabolic interventions.

An entangled metabolic cognition perspective may also help link findings of resting-state neurometabolic inefficiency to psychiatric symptoms, potentially related to the lived experience of being a self-in-context. One category of relevant psychiatric symptoms is the experience of “disembodiment” that may be present across a range of conditions and schizophrenia in particular (Fuchs and Schlimme, 2009). Symptoms of disembodiment include a disruption of self-recognition of bodily actions and ownership that undermines emotional expression and comprises social communication (Tschacher et al., 2017). While these findings have largely been interpreted with respect to neural dysfunction, findings of disembodiment in psychiatry may benefit from models that consider metabolism as playing a more than just a supporting role. For example, biopsychosocial models of cognition (Seery, 2011) as well as studies of cognitive effort (Westbrook and Braver, 2015) emphasize that information processing depends on the perception of cognitive resources and energetic availability. Despite robust relationships between predictive appraisal of cognitive resources and cardiovascular function (Seery, 2011), the effect of energetic resource distribution on brain function is less commonly studied. If the experience of “stress” is linked to changes in the dynamics of neurometabolic coupling (Elbau et al., 2018) similar changes may occur more broadly across emotional states or in psychiatric disorders whereby direct or indirect shifts in regionally available metabolism may modify neural activation. In this manner, stress may prove to be a particularly pronounced example of basic arousal and emotional phenomena, invoking metabolic work to meet the demands of anticipated behavior. The second-by-second time-scale of slow cortical potentials and the BOLD response is well matched to the intensity of emotional dynamics over time (Verduyn et al., 2009, 2015). More speculatively, studies of entangled neurometabolic coupling and invoked work may provide a framework to investigate the background “emotional feel” of the altered embodied experience associated with the suffering characteristic of psychiatric illness.

## Conclusion

We conclude that studies of information processing in biological systems must include special attention to embodiment, energetics and metabolism. We've shown that the significance of biological information must necessarily be processed in reference to a self-in-context. This contextual information must be reliably present in the background of ongoing neural activity, at both cellular and systems levels. Further, this context can be significantly modulated by metabolic activity. Emerging evidence suggests that metabolic and hemodynamic activity may be invoked to modulate self-in-context and that disruptions in this process may be undermined in psychiatric illness.

In our presentation of this model, we have glossed over important details relevant to cellular and systems neuroscience, such as the biophysics of neurovascular coupling and the relevance of dynamical systems in describing the far-from-equilibrium dynamics of neural processes. Future studies might examine interactions between EEG and BOLD activity from the perspective of nonlinear coupling dynamics (He, 2011), which may be of particular relevance because of the energetic cost/savings of far-from-equilibrium dynamics. Computational work has identified that metabolic constraints may be necessary for dynamical maintenance and state changes (Roberts et al., 2014; Burroni et al., 2017). Despite these important avenues for future work, significant limitations remain regarding how to model the effects of maintenance and invoked brain activity in neuroimaging paradigms. Moreover, new approaches will be needed to consider effects of background dynamics on traditionally evoked paradigms.

Ultimately, we suggest that the dynamics of neurometabolic coupling offers a new frontier for studying the biological foundations of cognition. Rather than viewing metabolism as a surrogate or passive support, it can be considered as an active signal in its own right. For this reason, neuroimaging paradigms, particularly resting-state fMRI that rely on changes in blood oxygen to infer neural stimulus processing and spontaneous cognition, are enhanced by including direct measures of neural activity from EEG. Despite the difficulty of implementation, we hope these technical and conceptual implications inspire further work integrating EEG with fMRI.

## Data availability statement

The original contributions presented in the study are included in the article/supplementary material, further inquiries can be directed to the corresponding author.

## Author contributions

MJ and TD developed the initial conceptual framework and theory. JF contributed to the sections on the resting-state paradigm and metabolism and embodied psychiatry. MJ wrote the first draft and designed and built Figures 1, 2. TD designed and built Figure 3. All authors contributed key observations that refined the conceptual framework, revised, and edited the manuscript.
